# Serum diagnosis of diffuse large B-cell lymphomas and further identification of response to therapy using SELDI-TOF-MS and tree analysis patterning

**DOI:** 10.1186/1471-2407-7-235

**Published:** 2007-12-29

**Authors:** Xing Zhang, Bo Wang, Xiao-shi Zhang, Zhi-ming Li, Zhong-zhen Guan, Wen-qi Jiang

**Affiliations:** 1 Department of Medical Oncology, Sun Yat-sen University Cancer Center, Guangzhou, 510060, China; 2 Department of Biotherapy, Sun Yat-sen University Cancer Center, Guangzhou, 510060, China; 3 State Key Laboratory of Oncology in Southern China, Sun Yat-sen University Cancer Center, Guangzhou, 510060, China

## Abstract

**Background:**

Currently, there are no satisfactory biomarkers available to screen for diffuse large B cell lymphoma (DLBCL) or to identify patients who do not benefit from standard anti-cancer therapies. In this study, we used serum proteomic mass spectra to identify potential serum biomarkers and biomarker patterns for detecting DLBCL and patient responses to therapy.

**Methods:**

The proteomic spectra of crude sera from 132 patients with DLBCL and 75 controls were performed by SELDI-TOF-MS and analyzed by Biomarker Patterns Software.

**Results:**

Nine peaks were considered as potential DLBCL discriminatory biomarkers. Four peaks were considered as biomarkers for predicting the patient response to standard therapy. The proteomic patterns achieved a sensitivity of 94% and a specificity of 94% for detecting DLBCL samples in the test set of 85 samples, and achieved a sensitivity of 94% and a specificity of 92% for detecting poor prognosis patients in the test set of 66 samples.

**Conclusion:**

These proteomic patterns and potential biomarkers are hoped to be useful in clinical applications for detecting DLBCL patients and predicting the response to therapy.

## Background

Diffuse large B-cell lymphoma (DLBCL), the most common subtype of non-Hodgkin lymphoma (NHL) in adults, is a potentially curable disease. Nonetheless, with currently available treatment options, long-term remission can only be achieved in about 50% of all diagnosed patients. Detecting cancers at their earliest stages will result in higher rates for curing the disease [1,2]. The application of new technologies for the earlier detection of DLBCL could have an important effect on public health, and to achieve this goal, specific and sensitive molecular markers are essential.

Each organ and tissue perfused by blood can contribute to modify or remove circulating proteins and peptides. Consequently, the serum proteome may reflect the abnormality or pathologic state of organs and tissues [3]. By using surface enhanced laser desorption/ionization time-of-flight mass spectrometry (SELDI-TOF-MS), Liotta *et al. *[4] identified proteomic patterns in serum that distinguished neoplastic disease from non-neoplastic disease within the ovary. This result yielded a sensitivity of 100%, a specificity of 95%, and a positive predictive value of 94%. Another study showed that the proteomic pattern correctly predicted 36 (95%; 95% confidence interval [CI] = 82–99%) of 38 patients with prostate cancer, while 177 (78%) of 228 patients were correctly classified as having benign conditions. For men with marginally elevated PSA levels, the specificity was 71% [3]. Other groups also used this approach successfully to diagnose ovarian, prostate [5-7], and breast cancers [8-10]. Mauvieux *et al.*[11] identified and characterized markers of interest in chronic B-cell malignancies. This study emphasized the usefulness of mass spectrometry studies in such malignancies. Lin *et al.*[12] identified proteins that may be involved in FL progression using SELDI. They rapidly identified a number of potential candidate proteins with specific regard to FL transformation. Their studies demonstrate the utility of SELDI-TOF-MS for the rapid discovery of differentially expressed proteins using femtomolar quantities of crude protein derived from biopsy material.

Although DLBCL is a curable disease, fewer than one-half of all diagnosed patients are cured with conventional chemotherapy. It is necessary to identify patients who do not benefit from standard treatment and should receive risk-adjusted therapies [13]. In 1993, the international prognostic index (IPI; age, performance status, stage, number of extranodal sites, and serum lactate dehydrogenase [LDH]) was proposed based on overall survival rates of 2031 adults of all ages with aggressive lymphomas who were treated in the United States, Canada, and Europe with doxorubicin-based chemotherapy with or without involved-field radiotherapy [14]. This system can be used to determine treatment and allow results to be compared among centers. IPI is the current gold standard parameter of prediction and it is mainly a clinical prognostic model developed to identify DLBCL patients who are unlikely to be cured with standard therapy. However, IPI is imperfect in its identification of high-risk patients for the intrinsic molecular heterogeneity in this disease [15]. Therefore, it is important to find serum biomarkers for distinguishing between good prognosis groups and poor prognosis groups. SELDI-TOF-MS is one of the currently used techniques to identify cancer biomarkers. SELDI profiling has been used successfully to differentiate ovarian, breast, prostate, and liver cancers from controls [9,10,16,17].

The aim of this study was to explore the application of serum SELDI proteomic patterns for distinguishing DLBCL patients from healthy individuals and distinguishing good prognosis patients from poor prognosis patients.

## Methods

### Patients and samples

Serum samples were collected from the Bank of Tumor Resource of patients, with prior consent from the donors, at the Cancer Center of Sun Yat-sen University. Diagnoses were confirmed by pathology and serum specimens were obtained before treatment. The study was approved by the Research Ethics Committee of the Cancer Center at Sun Yat-sen University. This study included 207 specimens, 132 samples of which were obtained from DLBCL patients and 75 samples which were from healthy individuals in the Cancer Center of Sun Yat-sen University during routine examinations.

The samples were separated into two groups during the process of detecting DLBCL. The training group consisted of 80 patients and 42 controls and the test group had 52 patients and 33 controls. The median age of the healthy controls was 45 years (range, 23–73 years). The median age of the cancer group patients was 52 years (range, 21–72 years). The clinical stage distribution of the 132 patients was as follows: stage I (n = 16); stage II (n = 56); stage III (n = 44); and stage IV (n = 16). There were 26 patients with an IPI of 0, 30 with an IPI of 1, 24 with an IPI of 2, 37 with an IPI of 3, and 15 with an IPI of 4.

The next level of categorization included the 132 DLBCL specimens in the study of the BPS (Biomarker Pattern software) algorithm to discriminate the poor prognosis group from the good prognosis group. The follow-up period from diagnosis was 36 to 48 months. Patients alive for more than 36 months from the time of diagnosis were classified as the good prognosis group and patients alive less than 36 months from the time of diagnosis were classified as the poor prognosis group. Eighty-three samples were obtained from the good prognosis patients and 49 samples were from the poor prognosis patients. The specimens were separated into two groups: 1) the training group, with 33 good prognosis patients and 33 poor prognosis patients and 2) the testing group with 50 good prognosis patients and 16 poor prognosis patients.

The next study was performed to discriminate the relapse group from the non-relapse group. Patients who relapsed for less than 36 months from the time of diagnosis were classified into the relapse group and patients who did not were classified into the poor prognosis group; 62 non-relapse patients and 70 relapse patients were included. The specimens were separated into two groups: 1) the training group with 33 non-relapse patients and 33 non-relapse patients and 2) the testing group with 29 non-relapse patients and 37 relapse patients.

All blood samples were obtained in the morning before food intake, aliquoted into 40 μl specimens, and stored at -80°C prior to running the assays.

### Proteomics Data Set

The protocol reported by Adam *et al. *(2002) which was used to classify SELDI-TOF MS spectra from 207 samples, was followed. In brief, 10 μl of each serum sample and 90 μl of a solution containing 0.5% CHAPS (Sigma, Inc., St. Louis, MO, USA) in phosphate-buffered saline (pH 7.4) were added to each well of a 96-well plate. The mixture was vortex-mixed at 4°C for 15 min, followed by the addition of 100 μl of Cibacron Blue 3GA (Sigma; prepared and balanced in 0.5% CHAPS three times). The plates were placed on a platform shaker at 4°C for 60 min. After centrifugation, the supernatant (40 μl) was then transferred onto the WCX2 chips so that each chip (8-spot format) held four tumorous and four healthy samples to rule out systematic error. All samples, including the training set, test set, and normal serum quality control (QC) sample were positioned randomly on the chips. The chips were placed in a bioprocessor (Ciphergen Biosystems, Inc.), which holds 12 chips and allows a larger volume of serum to be applied to each chip array. The samples were allowed to react with the surface of the WCX2 chip for 60 min at room temperature. The chips were then washed three times by gently shaking on a platform shaker at a speed of 700 rpm for 5 min with 200 μl of 20 mmol/L HEPES (pH 7.4), air dried, and crystallized by the addition of α-cyano-4-hydroxycinnamic acid (CHCA; Ciphergen Biosystems, Inc.). The chips were read on a protein biological system II (PBS-II) and a mass spectrometer reader (Ciphergen Biosystems, Inc.). The data were collected by averaging 140 laser shots with an intensity of 150, a detector sensitivity of 6, a peak mass of 30,000 Da, and an optimized range of 2,000–20,000 Da. The mass accuracy was calibrated to < 0.1% using the All-in-1 Peptide Molecular Mass Standard (Ciphergen Biosystems, Inc.). Each spectrum was composed of peak amplitude measurements at approximately 15,200 points, defined by a corresponding mass-to-charge ratio (M/Z) value.

### Bioinformatics and Biostatistics

Using Biomarker Wizard software (Ciphergen Biosystems, Inc.), we compiled all spectra. The qualified mass peaks (signal-to-noise ratio > 5) with a M/Z between 2000 and 20,000 were detected automatically. The peak clusters were completed with second-pass peak selection (signal-to-noise ratio > 2 within a 0.3% mass window), and the estimated peaks were added. The peak intensities were normalized to the total ion current of the M/Zbetween 2000 and 20,000 using Protein-Chip software, version 3.0 (Ciphergen Biosystems, Inc.).

### Decision tree classification

Construction of the decision tree classification algorithm was performed by Ciphergen Biomarker Pattern software, version 5.0. The classification tree split the data into two nodes, using one rule at a time, to form peak intensities. The splitting decisions in this case were based on the normalized intensity levels of the peaks from the SELDI protein expression profile. The process of splitting was continued until the terminal nodes were produced. After performing the V-fold cross validation 50, the accuracy of each classification tree was challenged with the blinded testing set.

### Statistical Analysis

A Bayesian approach was used to calculate the expected probabilities of each class in each terminal node. Comparison of relative peak intensity levels between groups was made using the Student's *t *test and in all cases, *P *< 0.05 was considered statistically significant. Specificity was calculated as the ratio of the number of non-cancer samples, good prognosis samples, or non-relapse samples correctly classified to the total number of non-cancer samples, good prognosis samples, or non-relapse samples, respectively. Sensitivity was calculated at the ratio of the number of correctly classified DLBCL samples, poor prognosis samples, or relapse patients to the total number of DLBCL samples, poor prognosis samples, or relapse samples.

## Results

### Identification of specific serum proteomic features

A total of four chip chemistries (hydrophobic surface, immobilized metal affinity capture, weak cation exchange [WCX], and strong anion exchange) were evaluated to investigate which provided the best serum profile. Our determinations revealed that the WCX chip provided the most discriminating pattern for constructing a decision tree.

Serum samples (n = 122; 42 controls and 80 cancers) in the training set were assayed by SELDI mass spectrometry. Another 85 samples (33 controls and 52 cancers) were selected for the blinded test set for the algorithm. The SELDI technology was particularly effective in resolving the low molecular weight (< 20 kDa) proteins and polypeptides. Peaks with a M/Z < 2 kDa were comprised mainly of ion noise from the matrix and were therefore excluded. Nine top-scored peaks (*P *< 0.01) at M/Zs of 2821, 2954, 3266, 4779, 5638, 5707, 5838, 5907, and 7975 were selected for analysis (Table 1). For separating the groups, the sensitivity was from 62% to 84% and specificity was from 73% to 85%. These nine representative peaks were higher in the tumor samples compared with the controls and considered to be potential biomarkers for discriminating DLBCL patients from non-cancerous patients. These representative spectra were shown in the Additional file 1, which showed nine protein biomarkers in serum for detection of DLBCL.

**Table 1:** Proteomic features showing significantly differences in expression by ProteinChip in detection of DLBCL.

Mass(Da)	p	Ctrl(mean)	Ctrl(SD)	Tumor(mean)	Tumor(SD)	Fold
2821.906	5.02E-06	2.614971	1.582986	4.566231	2.535257	1.746188
2954.257	5.51E-06	1.308963	0.767800	2.965317	1.236755	2.265394
3266.786	3.45E-07	1.218587	0.894500	2.726363	1.368996	2.237315
4779.803	6.56E-05	0.948255	0.563501	2.007074	1.242644	2.116597
5638.932	3.56E-06	21.96427	6.529237	40.96807	12.88758	1.865215
5707.424	1.32E-06	1.697549	0.592136	3.525848	0.978960	2.077023
5838.342	8.04E-07	2.149179	1.109202	4.132974	1.782941	1.923048
5907.698	4.44E-06	4.715597	2.001269	14.39084	4.695559	3.051754
7975.704	0.025325	4.392209	3.298483	10.10014	3.349020	2.299559

DLBCL: Diffuse large B-cell lymphomas; Mean and SD refer to the peak intensities

Of the 132 DLBCL serum samples, 66 samples, including 33 good prognosis samples and 33 poor prognosis samples, were chosen randomly for the learning set and 66 samples, including 50 good prognosis samples and 16 poor prognosis samples, were selected for the blinded test set, respectively. Four top-scored peaks (*P *< 0.05), at M/Zs of 4078, 4304, 5481, and 8608 were selected as potential discriminatory biomarkers for the good prognosis and poor prognosis cases (Table 2). For separating the groups, the sensitivity was from 61% to 80% and specificity was from 65% to 88%. All four peaks were higher in the good prognosis patients than in the poor prognosis patients. Five peaks at M/Zs of 2954, 4304, 4320, 5069, and 16093 were chosen for potential biomarkers for discriminating relapse patients from non-relapse patients (Table 3). Four peaks at M/Zs of 4304, 4320, 5069, and 16093 were higher in the non-relapse patients than in the relapse patients. For separating the groups, the sensitivity was from 60% to 74% and specificity was from 59% to 76%.

**Table 2:** Proteomic features showing differences in prediction of response to therapy by ProteinChip in DLBCL patients.

Mass(Da)	p	GP(mean)	GP(SD)	PP(mean)	PP (SD)	Fold
4078.883	0.01478	4.453288	2.181249	2.785609	1.187186	1.598677
4304.084	0.006578	8.614694	2.94989	3.437385	1.374539	2.506176
5481.569	0.018384	5.328566	2.653169	3.256677	1.239096	1.636197
8608.812	0.01478	4.453288	1.681249	2.785609	1.187186	1.598677

GP: Good prognosis; PP: Poor prognosis; Mean and SD refer to the peak intensities.

**Table 3:** Proteomic features showing significantly differences in prediction of relapse by ProteinChip in DLBCL patients.

Mass(Da)	p	NR(mean)	NR(SD)	RE(mean)	RE(SD)	Fold
2954.992	0.041855	1.707398	0.795678	3.394241	1.859434	1.987961
4304.379	0.001273	9.265967	2.843153	4.905272	2.405961	0.529386
4320.266	0.030246	2.983608	1.15555	1.239195	0.72511	0.415334
5069.691	0.045795	2.972709	1.139147	1.282858	0.734228	0.431545
16093.56	0.043788	2.891461	1.049451	1.364795	0.534483	0.472009

NR: Non-relapse group; RE: Relapse group; PFS: Progression free survival; Mean and SD refer to the peak intensities.

### Decision tree construction

Breiman *et al. *developed a decision tree (DT) model, which uses a variant of the classification and regression tree (CART) method. This method consists of two steps: 1) tree construction and 2) tree pruning [18,19]. In the tree construction process, the best predictor variables were identified with algorithms that divided the parent node sample into two child nodes. The decision tree classifies a particular pattern through a sequence of questions, beginning at the root node, and formulates the subsequent questions based upon the initial answers. This process is repeated until a terminal node is attained. At the end of the process, each terminal node contains a certain percentage of tumor samples. This percentage specifies the probability of a sample as being tumorous. If a terminal node contains the proportion of tumor sample, (p) > 50% (i.e., p > 0.5), then all the samples in this terminal are designated as tumor samples, and p is the probability value assigned to the entire sample in this terminal node. Similarly, samples are non-tumorous if the probability is < 0.5.

**Figure 1:**
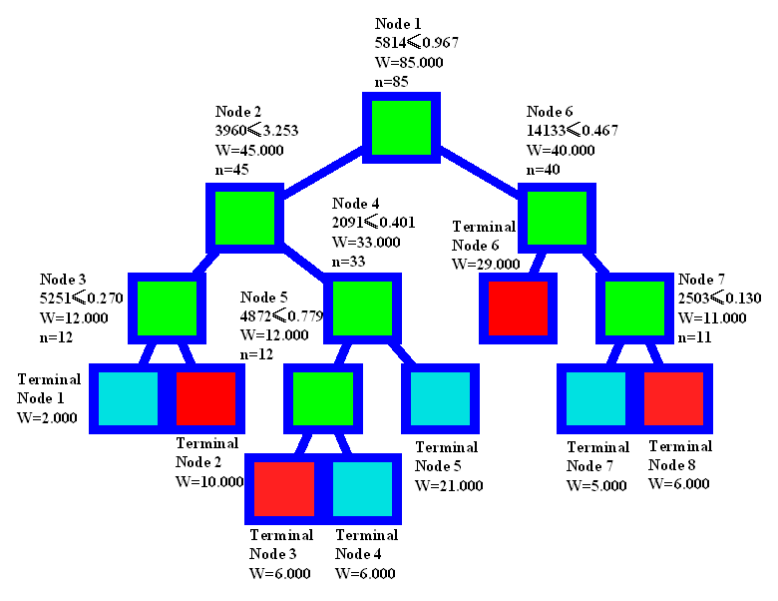
**Diagram of decision tree analysis pattern of classification of DLBCL *vs*. normal samples**. The root node (top), descendant nodes and the terminal nodes (Node 1–Node 7) are shown as squares. N represents the number of samples. The first number under the root and descendant nodes is the mass value followed by the peak intensity value. For example, the mass value under the root node is 5814 Da, and the intensity is 0.967. DLBCL: Diffuse large B-cell lymphomas.

Seven peaks at 2091, 2503, 3960, 4872, 5251, 5814, and 14,133 Da were selected by the BPS algorithm to discriminate DLBCL samples from control samples. Figure 1 illustrates the decision tree that was generated from the learning set to classify the two groups. The classification algorithm correctly predicted 98 % (41 of 42) and 99 % (79 of 80) of the samples from the control and the DLBCL groups, respectively. Analyses of the spectra from the 85 testing samples showed that the classification algorithm correctly predicted 94% (80 of 85) of all of the samples, with 94% (49 of 52) of DLBCL samples and 94% (31 of 33) of the control samples. The specificity was 94% and the sensitivity was 94%. Most importantly, 16 cases of stage I patients were all identified correctly (Table 4). The representative spectra in the decision tree for the selected diagnostic peaks were shown in the Additional file 2, which showed seven serum peaks in mass pattern for diagnosis of SELDI.

**Table 4:** The sensitivity of the SELDI marker pattern in detecting different DLBCL stages.

Variables Clinical stages	n	Correct cases	Error cases	Sensitivity (%)	P value
I	16	16	0	100.00	<0.05
II	56	54	2	96.43	<0.05
III	44	40	4	90.91	<0.05
IV	16	15	1	93.75	<0.05

DLBCL: Diffuse large B-cell lymphomas

**Figure 2:**
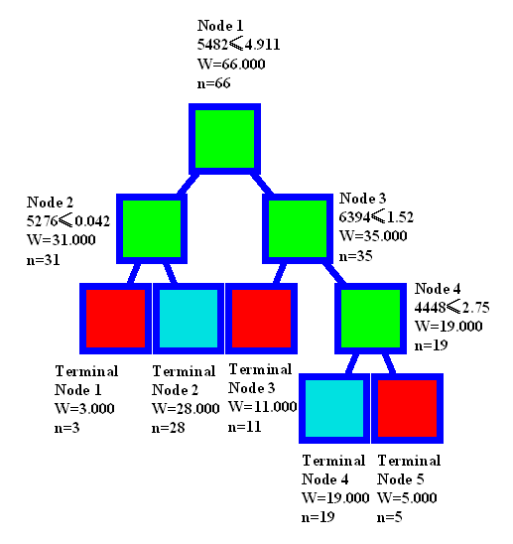
**Diagram of decision tree analysis pattern of classification of poor prognosis *vs*. good prognosis samples**. The root node (top), descendant nodes and the terminal nodes (Node 1–Node 4) are shown as squares. N represents the number of samples. The first number under the root and descendant nodes is the mass value followed by the peak intensity value. For example, the mass value under the root node is 5482 Da, and the intensity is 4.911.

To discriminate the poor prognosis group from the good prognosis group, four peaks at 4448, 5276, 5482, and 6394 Da were chosen by the BPS algorithm. The decision tree to classify the two groups is shown in Figure 2. In the training set, the classification algorithm correctly predicted 100% of the samples from the good prognosis group and 100% of the samples from the poor prognosis group. Analyses of the spectra in the test set showed that the classification algorithm correctly predicted 92 % (61 of 66) of all of the samples with 92 % (46 of 50) of the good prognosis group and 94 % (15 of 16) of the poor prognosis group. The specificity was 92 % and the sensitivity was 94 %.

**Figure 3:**
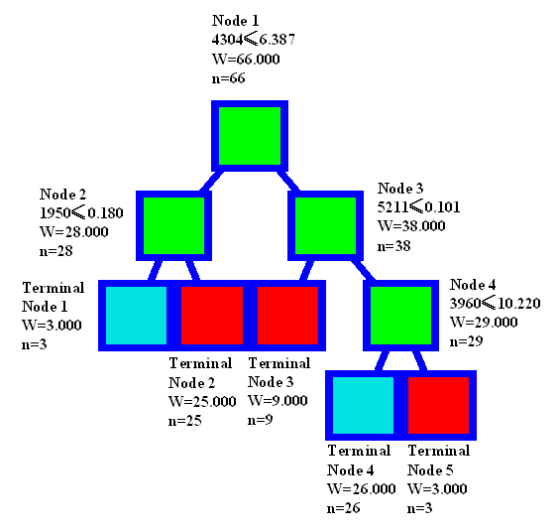
**Diagram of decision tree analysis pattern of classification of relapse *vs*. non-relapse samples**. The root node (top), descendant nodes and the terminal nodes (Node 1–Node 4) are shown as squares. N represents the number of samples. The first number under the root and descendant nodes is the mass value followed by the peak intensity value. For example, the mass value under the root node is 4304 Da, and the intensity is 6.387.

Four peaks at 1950, 3960, 4304, and 5211 Da were chosen for patterns to discriminate the relapse group from the non-relapse group. Figure 3 showes the decision tree to classify these two groups. In the training set, the classification algorithm correctly predicted 100% of the samples for the non-relapse group and 100% of the samples for the relapse group. In the testing analyses, the classification algorithm correctly predicted 91% (60 of 66) of all of the samples with 90% (26 of 29) of the non-relapse group and 92% (34 of 37) of the relapse group. The specificity was 90% and the sensitivity was 92%.

### Reproducibility and precision

To assess the precision and the accuracy of the proteomic data in our analyses, we employed external calibration standards using the All-in-1 Peptide Molecular Mass Standard (Ciphergen Biosystems, Inc.), which allowed us to achieve a mass accuracy of approximately 1 Da in 10,000. To confirm the reproducibility of SELDI spectra in our study, namely, the intensity from array-to-array on a single chip (intra-assay) and between chips (inter-assay) was determined using the pooled normal serum quality control (QC) sample. Ten selected M/Z peaks were randomly selected and compared to calculate the coefficient of variance. The intra-assay analyses were performed in triplicate and the inter-assay analyses were performed on three different days. The intra- and inter-assay mean CV for the normalized intensity were 10% and 14%, respectively.

### IPI

The IPI was defined as a positive criterion to identify DLBCL patients who are unlikely to be cured with standard therapy. Patients were grouped into two subgroups: 1) an IPI of 0–2 and 2) an IPI of 3–4. Patients with an IPI of 0–2 were considered as patients with a good prognosis and patients with an IPI of 3–4 were considered as patients with a poor prognosis. For predicting DLBCL patients with a poor prognosis, the sensitivity of IPI was 61% (30/49) and the specificity was 80% (66/83), while the specificity of the SELDI classification model was 92 % and the sensitivity was 94 %. The SELDI classification model predicted the response significantly better than the conventional IPI.

## Discussion

For the majority of patients, DLBCL is a systemic disease at the time of diagnosis. At the completion of the initial staging evaluation, stages II, III, or IV disease are documented in approximately 75% of all DLBCL patients [2]. Thus, the search for new early serum diagnostic markers of DLBCL will be important for the detection of early stage DLBCL patients who have a longer survival rate. The recent development of surface-enhanced laser desorption/ionization technology, based on improved methods for the chemical preparation of absorptive surfaces and their use for solid-state mass spectrometry, allows high-throughput protein analysis of crude biologic samples [16]. Using these techniques in combination promises a working approach for identifying potential DLBCL biomarkers for early-stage diagnosis and treatment.

Low-molecular-weight serum protein profiling may reflect the pathologic state of organs and aid in the early detection of cancer. Furthermore, MALDI-TOF and SELDI-TOF mass spectrometry can profile proteins in this range. These profiles can contain thousands of data points, necessitating sophisticated analytical tools. Bioinformatics has been used to study physiologic outcomes and cluster gene microarrays [6].

The application of proteomics to the analysis of the human prostate could potentially uncover useful biomarkers. SELDI offers the advantages of rapid, high throughput screening using small volumes of clinical samples, and includes rapidity and reproducibility in the screening of protein expression profiles (also known as 'phenomic fingerprints'). However, there are no published data on the use of this technique coupled with a decision tree algorithm in studies of DLBCL protein profiles. In this study, we demonstrated that SELDI profiling of serum significantly, accurately, and reproducibly distinguished patients with DLBCL from healthy controls. The pattern also had a sensitivity of 100% for detecting stage I DLBCL patients, suggesting that the pattern may be better suited for the early detection of DLBCL. Similar to previous report that the SELDI decision tree captured more of the "early" (grade I and II) bladder cancer [20].

DLBCL is the most common type of NHL and accounts for approximately one-third of the total number of adult NHL patients. Although it represents a curable disease, fewer than one-half of the patients are cured with conventional chemotherapy. Identification of patients who do not benefit from current treatment may constitute the basis for risk-adjusted therapies for DLBCL. Therefore, it is important to develop a method for identifying patients who may be candidates for investigational approaches, and to distinguish high-risk patients from patients who benefit from the standard therapy. The disease free survival (DFS) and overall survival (OS) are the best end-points for predicting the prognosis of DLBCL patients. Three-year DFS and OS were observed in our study. The decision tree to classify two groups by the BPS algorithm correctly predicted that 92% of all of the sampled cases who could or could not benefit from the anti- DLBCL standard therapy.

However, the peaks in biomarkers and tree pattern were not the same, which could be explained by the fact that biomarkers had been identified as a single peak which showed the highest discrepancy between two groups, while the patterns stressed synergistic effects of peaks in-group. Moreover, peaks in the pattern still showed a significant difference in two groups, but the degree of difference was less than those in biomarkers.

However, in our study, the tree patterns from BPS indeed showed higher sensitivity and specificity than single biomarker or biomarker combination, they represented the highest discrepancy between two groups and may be good candidates for further protein identification analysis. Using SELDI system to analyze the urine samples from transitional cell carcinoma (TCC) from bladder and control samples, Vlahou *et al.*[21,22]identified several novel biomarkers for TCC diagnosis. Their further works identified α-defensin as one of the biomarkers, and expression of α-defensin peptides in bladder cancer cells increased with tumor invasiveness. The above experiments paved the way for further identification of single biomarker. In the future we will broaden the number of samples to testify these biomarkers and their combination.

The IPI, a clinical predictor for overall survival (OS), has been the primary prognostic model used in the management of patients with DLBCL. The subdivision of patients according to the number of prognostic factors into low risk (none or one factor), low-intermediate risk (two factors), high-intermediate risk (three factors), or high risk (four or five factors) with predicted 5-year OS values of 73%, 51%, 43%, and 26%, respectively, rapidly became the most widely used and accepted prognostic model for intermediate-grade lymphoma [14]. However, in all the clinical models, including the IPI index, there was marked residual heterogeneity in outcome, which was reflected by considerably variable survival of patients with identical prognostic scores. The latter was explained by the marked genetic and molecular heterogeneity that underlies disease aggressiveness and tumor progression, and led to evaluation of molecular and genetic markers associated with a patient's survival. SELDI biomarker and patterns could complement genetic and molecular heterogeneity which contribute to poor prognosis [23]. In our study, patients with an IPI of 0–2 were grouped together because they enjoyed dramatically better progression-free, cause-specific, and overall survival rates than those with an IPI of 3–4 [24]. Finally, the SELDI classification model was significantly better than the conventional IPI in the sensitivity and specificity of prediction.

There is a great need to discover novel biomarkers and translate them into routine clinical use. However, initial enthusiasm about these new technologies has been somewhat tempered by questions on method reproducibility. It has been reported that serum proteomic patterns obtained by the SELDI-TOF technique may not be reproducible and that the discriminatory peaks are not consistent, either within a group or among groups of investigators, for the same type of cancer [25-27].

By analysis of the publicly available data posted by Petricoin and coworkers [3,4], Diamandis [28,29] raised major concerns about the reproducibility of the SELDI-based approaches, whereas the concerns raised by Sorace and Zahn [30] and Baggerly *et al.*[31] was the bias of study design. There is also a great deal of controversy as to whether the use of high-throughput proteomic techniques, such as SELDI, can improve the early detection of cancer. The alternative hypothesis is that these differences between cancer and control groups are not due to the presence of cancer, but to something else. Possible confounders could include: 1) variability in sample collection, processing, and storage; 2) baseline characteristics of study subjects; 3) inappropriate statistical design; and 4) variations in mass spectrometer stability and protein chip performance. In addition, it is not known whether proteomic patterns differ between plasma and serum, or how they are influenced by lipemia, icterus, the number of freeze/thaw cycles the sample underwent, the sample's length of storage, or the subject's menstrual cycle, nutritional status, or drug use [29].

However, several groups have reported good reproducibility by improving the sample preparation methods [32,33]. Diamandis [29] raised concern that the "discriminating peaks are not consistent either within a group or among groups of individuals." The report of Semmes may be helpful to reconsider the comments of Diamandis [29]. Semmes clearly showed that the same three diagnostic peaks, at least the first strong diagnostic peak, were identified at multiple sites and were effective at differentiating case/control samples at all sites. These results demonstrated that the "between-laboratory" reproducibility of SELDI-TOF-MS serum profiling approaches that of "within-laboratory" reproducibility, as determined by measuring discrete M/Z peaks over time and across laboratories [34].

The limitations do not inhibit the application of mass spectrometry as an analytic tool or to other proteomic approaches used to identify proteins in serum or other biological fluids. Scientific skepticism and debate are essential to the progress of science. Analysis of poor quality, noise-laden protein expression profiles, however, will likely lead to results lacking biological relevance. Therefore, quality assessment of the protein expression profiles and determination of reproducibility of SELDI-TOF MS experiments and profiles prior to data analysis is of critical importance [34]. Low quality spectra should be identified and eliminated from analysis to ensure the reliability of biomarkers and the associated patterns discovered during analysis [35].

In our study, serum was collected in the morning before food intake, which helped to avoid the effects of lipemia and food. Serum was stored at -80°C and was not thawed until analysis to evade protein instability due to freezing and thawing. The pretreatment of serum followed the standard methodology of several references and the manual from Ciphergen Biosystems, Inc. Samples were randomly added on the chips to shun bias. The proteomics data set, decision tree classification, bioinformatic analysis, and biostatistics were performed by software from Ciphergen Biosystems, Inc. (Biomarker Wizard software and Ciphergen Biomarker Pattern software) according to several references and standard manuals. The mass range from 2000 to 20,000 Da was selected for analysis because this range contained the majority of the resolved protein/peptides. The molecular masses from 0 to 2000 Da were eliminated from analysis because this area contains adducts and artifacts of the EAM and possibly other chemical contaminants. The intra- and inter-assay mean CV for the normalized intensity in our study was below 15% and confirm the reproducibility. We used a testing set (the blinded set) to testify peaks and patterns from the training test, which validated the reproducibility. In the future, we will use another set of samples to verify the results and confirm the reproducibility.

The identity of the discriminating proteins by purification, identification, and characterization is currently under investigation. Revealing their identities will be essential for understanding the biological role of these peptide/proteins in the oncogenesis of DLBCL, potentially leading to novel therapeutic targets. Moreover, identifying these protein candidates will be essential for producing antibodies for developing classical immunoassays, similar to the quantitation techniques of PSA, prostate-specific membrane antigens.

## Conclusion

We found a proteomic archetype in serum that could distinguish DLBCL from non-tumorous control individuals and discriminate the poor prognosis group from the good prognosis group with high accuracy. The efficacy of screening early DLBCL and responsive patients would be increased with these proteomic patterns.

## Competing interests

The author(s) declare that they have no competing interests.

## Authors' contributions

XZ performed the SELDI-TOF-MS experiments and drafted the manuscript. BW participated in the SELDI-TOF-MS experiments. XSZ participated in the analysis and interpretation of SELDI-TOF-MS data. ZL participated in the follow-up of patients. ZG participated in the design of the study and revising the manuscript. WJ conceived of the study, and participated in its design and coordination. All authors read and approved the final manuscript.

## Pre-publication history

The pre-publication history for this paper can be accessed here:



## Supplementary Material

Additional file 1 **Supplemental figure 1 showing nine protein biomarkers in serum for detection of DLBCL**. Mass spectra of serum samples from two different DLBCL patients (Tumor1 and Tumor2) and two nontumor control (Ctrl-1 and Ctrl-2). The average molecular mass of the nine proteins identified to be unique or overexpressed in the tumor specimens are: 2821 Da, 2954 Da, 3266 Da, 4779 Da, 5638 Da, 5707 Da, 5838 Da, 5907 Da, and 7975 Da (arrow). Click here for file

Additional file 2 **Supplemental figure 2 showing seven serum peaks in mass pattern for diagnosis of SELDI**. Mass spectra of serum samples from two different DLBCL patients (Tumor1 and Tumor2) and two nontumor control (Ctrl-1 and Ctrl-2). The average molecular mass of the four proteins identified to be unique or overexpressed in the Ctrl specimens are: 2091 Da, 3960 Da, 4872 Da, and 14133 Da; the three DLBCL-associated proteins as listed in tumor specimens: 2503 Da, 5251 Da, and 5814 Da (arrow). Click here for file

## References

[B1] Wu G, Keating A (2006). Biomarkers of potential prognostic significance in diffuse large B-cell lymphoma. Cancer.

[B2] Abramson JS, Shipp MA (2005). Advances in the biology and therapy of diffuse large B-cell lymphoma: moving toward a molecularly targeted approach. Blood.

[B3] Petricoin EF, Ornstein DK, Paweletz CP, Ardekani A, Hackett PS, Hitt BA, Velassco A, Trucco C, Wiegand L, Wood K, Simone CB, Levine PJ, Linehan WM, Emmert-Buck MR, Steinberg SM, Kohn EC, Liotta LA (2002). Serum proteomic patterns for detection of prostate cancer. J Natl Cancer Inst.

[B4] Petricoin EF, Ardekani AM, Hitt BA, Levine PJ, Fusaro VA, Steinberg SM, Mills GB, Simone C, Fishman DA, Kohn EC, Liotta LA (2002). Use of proteomic patterns in serum to identify ovarian cancer. Lancet.

[B5] Walsh PC (2003). Serum proteomic patterns for detection of prostate cancer. J Urol.

[B6] Banez LL, Prasanna P, Sun L, Ali A, Zou Z, Adam BL, McLeod DG, Moul JW, Srivastava S (2003). Diagnostic potential of serum proteomic patterns in prostate cancer. J Urol.

[B7] Adam BL, Qu Y, Davis JW, Ward MD, Clements MA, Cazares LH, Semmes OJ, Schellhammer PF, Yasui Y, Feng Z, Wright GL (2002). Serum protein fingerprinting coupled with a pattern-matching algorithm distinguishes prostate cancer from benign prostate hyperplasia and healthy men. Cancer Res.

[B8] Wright GL (2002). SELDI proteinchip MS: a platform for biomarker discovery and cancer diagnosis. Expert Rev Mol Diagn.

[B9] Wulfkuhle JD, McLean KC, Paweletz CP, Sgroi DC, Trock BJ, Steeg PS, Petricoin EF (2001). New approaches to proteomic analysis of breast cancer. Proteomics.

[B10] Paweletz CP, Trock B, Pennanen M, Tsangaris T, Magnant C, Liotta LA, Petricoin EF (2001). Proteomic patterns of nipple aspirate fluids obtained by SELDI-TOF: potential for new biomarkers to aid in the diagnosis of breast cancer. Dis Markers.

[B11] Miguet L, Bogumil R, Decloquement P, Herbrecht R, Potier N, Mauvieux L, Van Dorsselaer A (2006). Discovery and identification of potential biomarkers in a prospective study of chronic lymphoid malignancies using SELDI-TOF-MS. J Proteome Res.

[B12] Lin Z, Jenson SD, Lim MS, Elenitoba-Johnson KS (2004). Application of SELDI-TOF mass spectrometry for the identification of differentially expressed proteins in transformed follicular lymphoma. Mod Pathol.

[B13] Lossos IS (2005). Molecular pathogenesis of diffuse large B-cell lymphoma. J Clin Oncol.

[B14] (1993). A predictive model for aggressive non-Hodgkin's lymphoma. The International Non-Hodgkin's Lymphoma Prognostic Factors Project. N Engl J Med.

[B15] Shipp MA, Ross KN, Tamayo P, Weng AP, Kutok JL, Aguiar RC, Gaasenbeek M, Angelo M, Reich M, Pinkus GS, Ray TS, Koval MA, Last KW, Norton A, Lister TA, Mesirov J, Neuberg DS, Lander ES, Aster JC, Golub TR (2002). Diffuse large B-cell lymphoma outcome prediction by gene-expression profiling and supervised machine learning. Nat Med.

[B16] Zhang H, Kong B, Qu X, Jia L, Deng B, Yang Q (2006). Biomarker discovery for ovarian cancer using SELDI-TOF-MS. Gynecol Oncol.

[B17] Issaq HJ, Veenstra TD, Conrads TP, Felschow D (2002). The SELDI-TOF MS approach to proteomics: protein profiling and biomarker identification. Biochem Biophys Res Commun.

[B18] Breiman L (1999). Random Forests. Technical Report
567..

[B19] P. C, Breiman L, Friedman J, Olshen R, Stone C, Steinberg D (1995). CART: Classification
and Regression Trees..

[B20] Vlahou A, Giannopoulos A, Gregory BW, Manousakas T, Kondylis FI, Wilson LL, Schellhammer PF, Wright GL, Semmes OJ (2004). Protein profiling in urine for the diagnosis of bladder cancer. Clin Chem.

[B21] Holterman DA, Diaz JI, Blackmore PF, Davis JW, Schellhammer PF, Corica A, Semmes OJ, Vlahou A (2006). Overexpression of alpha-defensin is associated with bladder cancer invasiveness. Urol Oncol.

[B22] Vlahou A, Schellhammer PF, Mendrinos S, Patel K, Kondylis FI, Gong L, Nasim S, Wright Jr GL (2001). Development of a novel proteomic approach for the detection of transitional cell carcinoma of the bladder in urine. Am J Pathol.

[B23] Lossos IS, Morgensztern D (2006). Prognostic biomarkers in diffuse large B-cell lymphoma. J Clin Oncol.

[B24] Wilder RB, Rodriguez MA, Medeiros LJ, Tucker SL, Ha CS, Romaguera JE, Pro B, Hess MA, Cabanillas F, Cox JD (2002). International prognostic index-based outcomes for diffuse large B-cell lymphomas. Cancer.

[B25] Diamandis EP (2004). Mass spectrometry as a diagnostic and a cancer biomarker discovery tool: opportunities and potential limitations. Mol Cell Proteomics.

[B26] Diamandis EP (2006). Serum proteomic profiling by matrix-assisted laser desorption-ionization time-of-flight mass spectrometry for cancer diagnosis: next steps. Cancer Res.

[B27] Baggerly KA, Morris JS, Edmonson SR, Coombes KR (2005). Signal in noise: evaluating reported reproducibility of serum proteomic tests for ovarian cancer. J Natl Cancer Inst.

[B28] Diamandis EP (2003). Re: Serum proteomic patterns for detection of prostate cancer. J Natl Cancer Inst.

[B29] Diamandis EP (2004). Analysis of serum proteomic patterns for early cancer diagnosis: drawing attention to potential problems. J Natl Cancer Inst.

[B30] Sorace JM, Zhan M (2003). A data review and re-assessment of ovarian cancer serum proteomic profiling. BMC Bioinformatics.

[B31] Baggerly KA, Morris JS, Coombes KR (2004). Reproducibility of SELDI-TOF protein patterns in serum: comparing datasets from different experiments. Bioinformatics.

[B32] Marshall J, Kupchak P, Zhu W, Yantha J, Vrees T, Furesz S, Jacks K, Smith C, Kireeva I, Zhang R, Takahashi M, Stanton E, Jackowski G (2003). Processing of serum proteins underlies the mass spectral fingerprinting of myocardial infarction. J Proteome Res.

[B33] van der Merwe DE, Oikonomopoulou K, Marshall J, Diamandis EP (2007). Mass spectrometry: uncovering the cancer proteome for diagnostics. Adv Cancer Res.

[B34] Semmes OJ, Feng Z, Adam BL, Banez LL, Bigbee WL, Campos D, Cazares LH, Chan DW, Grizzle WE, Izbicka E, Kagan J, Malik G, McLerran D, Moul JW, Partin A, Prasanna P, Rosenzweig J, Sokoll LJ, Srivastava S, Srivastava S, Thompson I, Welsh MJ, White N, Winget M, Yasui Y, Zhang Z, Zhu L (2005). Evaluation of serum protein profiling by surface-enhanced laser desorption/ionization time-of-flight mass spectrometry for the detection of prostate cancer: I. Assessment of platform reproducibility. Clin Chem.

[B35] Hong H, Dragan Y, Epstein J, Teitel C, Chen B, Xie Q, Fang H, Shi L, Perkins R, Tong W (2005). Quality control and quality assessment of data from surface-enhanced laser desorption/ionization (SELDI) time-of flight (TOF) mass spectrometry (MS). BMC Bioinformatics.

