# Supplementary figures and images for: Serum diagnosis of diffuse large B-cell lymphomas and further identification of response to therapy using SELDI-TOF-MS and tree analysis patterning
Source: BMC Cancer. 2007 Dec 29;7:235. doi: 10.1186/1471-2407-7-235 (PMC2242801; doi:10.1186/1471-2407-7-235)

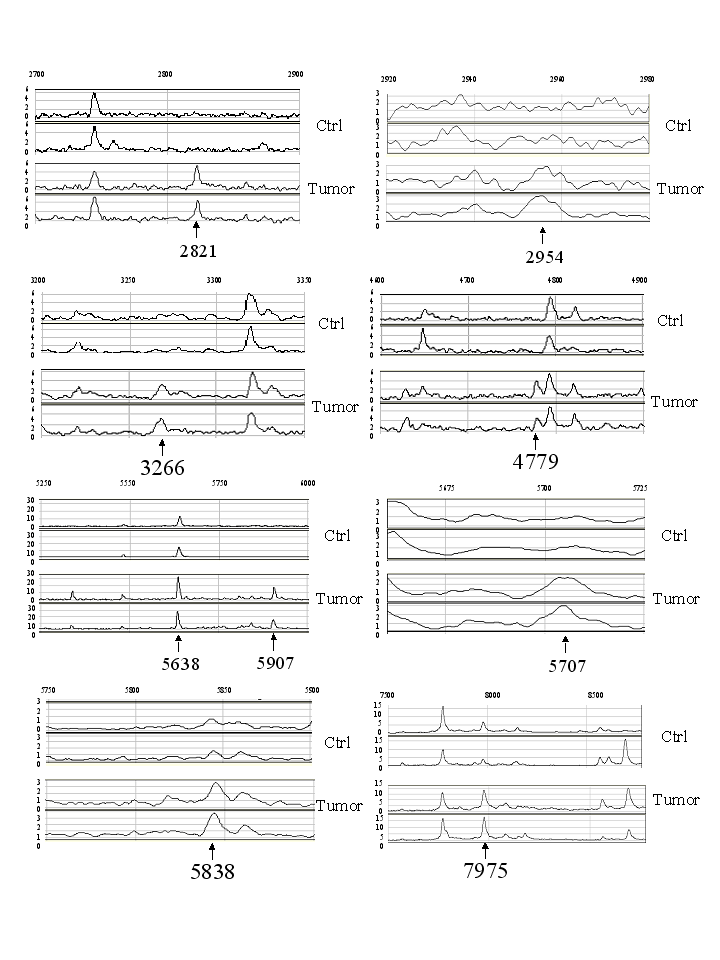

Supplement: Additional file 1 — Supplemental figure 1 showing nine protein biomarkers in serum for detection of DLBCL. Mass spectra of serum samples from two different DLBCL patients (Tumor1 and Tumor2) and two nontumor control (Ctrl-1 and Ctrl-2). The average molecular mass of the nine proteins identified to be unique or overexpressed in the tumor specimens are: 2821 Da, 2954 Da, 3266 Da, 4779 Da, 5638 Da, 5707 Da, 5838 Da, 5907 Da, and 7975 Da (arrow). [file 1471-2407-7-235-S1.TIFF]

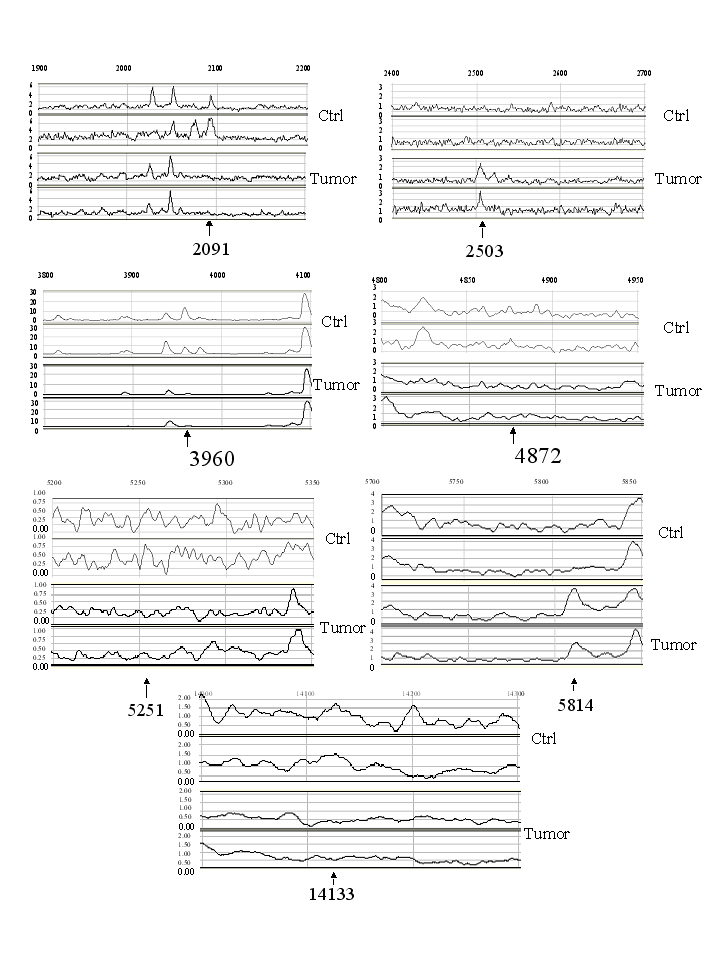

Supplement: Additional file 2 — Supplemental figure 2 showing seven serum peaks in mass pattern for diagnosis of SELDI. Mass spectra of serum samples from two different DLBCL patients (Tumor1 and Tumor2) and two nontumor control (Ctrl-1 and Ctrl-2). The average molecular mass of the four proteins identified to be unique or overexpressed in the Ctrl specimens are: 2091 Da, 3960 Da, 4872 Da, and 14133 Da; the three DLBCL-associated proteins as listed in tumor specimens: 2503 Da, 5251 Da, and 5814 Da (arrow). [file 1471-2407-7-235-S2.TIFF]
